# Light as a master switch: a critical review of photonanozymes from design to applications

**DOI:** 10.1007/s44307-026-00138-3

**Published:** 2026-10-13

**Authors:** Faria Fatima, Syed Saqib Ali Zaidi, Yang Guang, He Huang, Xiao-Man Sun

**Affiliations:** 1 School of Food Science and Pharmaceutical Engineering, Nanjing Normal University, 2 Xuelin Road, Qixia District, Nanjing, 210023 China; 2 Shenzhen Key Laboratory for Nano-Biosensing Technology, Research Center for Biosensor and Nanotheranostic, Guangdong Key Laboratory of Biomedical Measurements and Ultrasound Imaging, School of Biomedical Engineering, Shenzhen University, Shenzhen, 518060 China

**Keywords:** Photonanozymes, Light-responsiveness, Enzyme-like activity, Defects engineering, Photocatalysis

## Abstract

Despite the widespread industrial applications of nanozymes, their usage is often limited by mechanistic factors. They lack reaction control with catalyst fouling, which limits catalytic turnover and makes nanozyme recovery an energy-intensive process at large scale. These mechanistic challenges have been addressed by the evolution of photoresponsiveness into nanozymes called as photonanozymes (PNZs). This research is focused to critically assess the architecure of photonanozymes leading to photoresponsivness. A three domain framework deconstructing photoresponsive mechanism with research road map to guided subsequent investigation constitutes the distinctive contribution of this review. The photoresponsive architechture elucidates the mechanism of light gated catalysis governed by wavelength selectivity and spatiotemporal precison. Thereby, expanding the enzyme mimicking catalysis and its applciations in environmental remediation, theragnostics, cancer therapy, biosensing and biofuel synthesis. Rather than merely describing the operating principles of photonanozymes, rational foundation is build up which determines future design. Challenges impeding photocatalytic mechanisms are critically examined including limited wavelength selectivity, insufficient opertional stability and difficulty of achieving reproducible and scalable synthesis. Presented review strategizes a forward-looking roadmap centered around robust PNZ design, standardized performance, and translation of light-programmable catalysis from laboratory proof-of-concept to practical technology. Through a rigorous and comprehensive examination of inherent complexities, it frames PNZs as a transformative step towards programmable, self-healing nanomaterials with controlled implications, serving as an invaluable resource for those seeking orientation within photocatalysis. Furthermore, it reaches beyond its immediate boundaries, presenting new ideas, evidence-based conclusions, and prospective directions intended to influence the course of future investigations.

## Introduction

Photonanozymes are a class of nanozymes performing enzyme-like catalytic activity upon exposure to visible photons. They are activated by specific light photons,incorporating the programmable catalytic attributes of natural enzymes which can be customized and sustained via nanoscale photochemistry. Their light dependent activation distinguishes them from conventional nanozymes having an always-on mode of catalysis governed by the fixed chemical environment. This drawback of nanozymes creates a challenging situation through the production of undesired reactive oxygen species (ROS) and off-target effects in complex catalytic systems. Contrary to this, photonanozymes enable precise, on-demand activation. Their activity is confined to a particular area with a regulated activity potential mediated by light illumination. Hence, maximizing the efficiency and minimizing the undesired damage. Intensity, wavelength,or pulsing of light determines their reaction rate and substrate selectivity, which broadens their applications in sensing, theragnostics, and environmental remediation (Chakravorty and Roy 2024). Photonanozymes follow two mechanistic approaches to perform catalysis, i.e., intrinsic enzyme-like catalytic mechanisms and photoactivity mechanisms.

Along with photon activated catalytic potential, photonanozymes possess intrinsic enzyme-like activity. They follow a similar mechanism of action to nanozymes involving electron transfer (ET), substrate binding, and catalysis. The light-responsive activity of photonanozymes begins with the absorption of high-energy photons which excite the electrons through coherent oscillation. Hence, causing light absorption and heating. This is the photothermal conversion which eventually leads to the photocatalytic mechanisms. Nanorod photonanozymes of TiO₂ show two distinct enzyme-like activities in the presence and absence of light. In the dark, they exhibit catalase-like activity by decomposing H₂O₂ into O₂ and H₂O. Whereas, in the presence of visible illumination, they present peroxidase-like activity by using H₂O₂ to oxidize the chromogenic substrate 3,3′,5,5′-tetramethylbenzidine (TMB) (Huang et al. 2019; Leng et al. 2025; Liang and Yan 2019; Lin et al. 2022; Wang et al. 2019).

The dynamic relationship between photoactivity and enzyme-like activity of photonanozymes operates in three forms, i.e., photo enhanced enzyme like activity, photo activated enzyme like activity, and photo synergistic enzyme like activity (YongLe He et al. 2025a; C. Wang et al. 2021b). The influence of light has notable effects on photonanozymes in the form of activation or enhancement of their activity which elaborates their complex and diverse catalytic behavior. The operating principle of photoinduced activity of photonanozymes is as follows.

## Operating principle

**Fig. 1:**
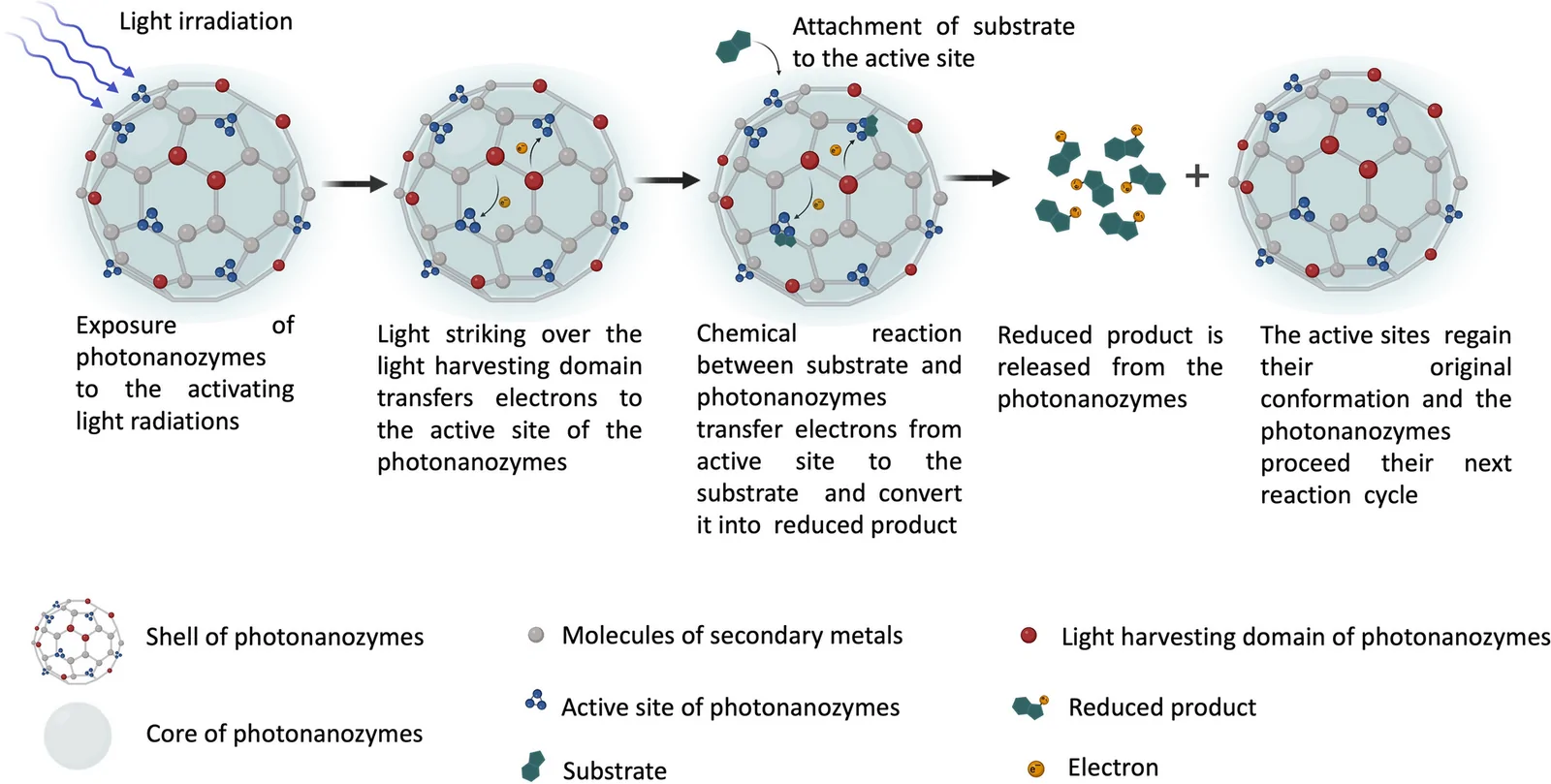
Light mediated catalysis: Operating principle of photonanozymes from light irradiation over PNZs to the final product release from the active site of PNZs

The operating principles of photonanozymes are based on three functional domains: (i) a light harvesting domain which produces high energy carriers/states upon irradiation like plasmonic metals, semiconductors, organic dyes or upconversion core, (ii) an enzyme mimetic active site which generates chemical outcomes from photoenergy such as vacancy, heteroatom dopant, single atom or molecular co-catalyst, (iii) a nano environment which sets up substrates and prevents electron–hole recombination. The combinatorial effect of these domains represents enzyme-like activity which follows Michaelis–Menten kinetics. Their Km and Vmax can be adjusted by photon flux rather than along with pH, temperature, time and substrate concentration as shown in Fig. 1 (Wang et al. 2020; Zhang and Liu 2020).

### Light-harvesting domain (LHD)

**Fig. 2:**
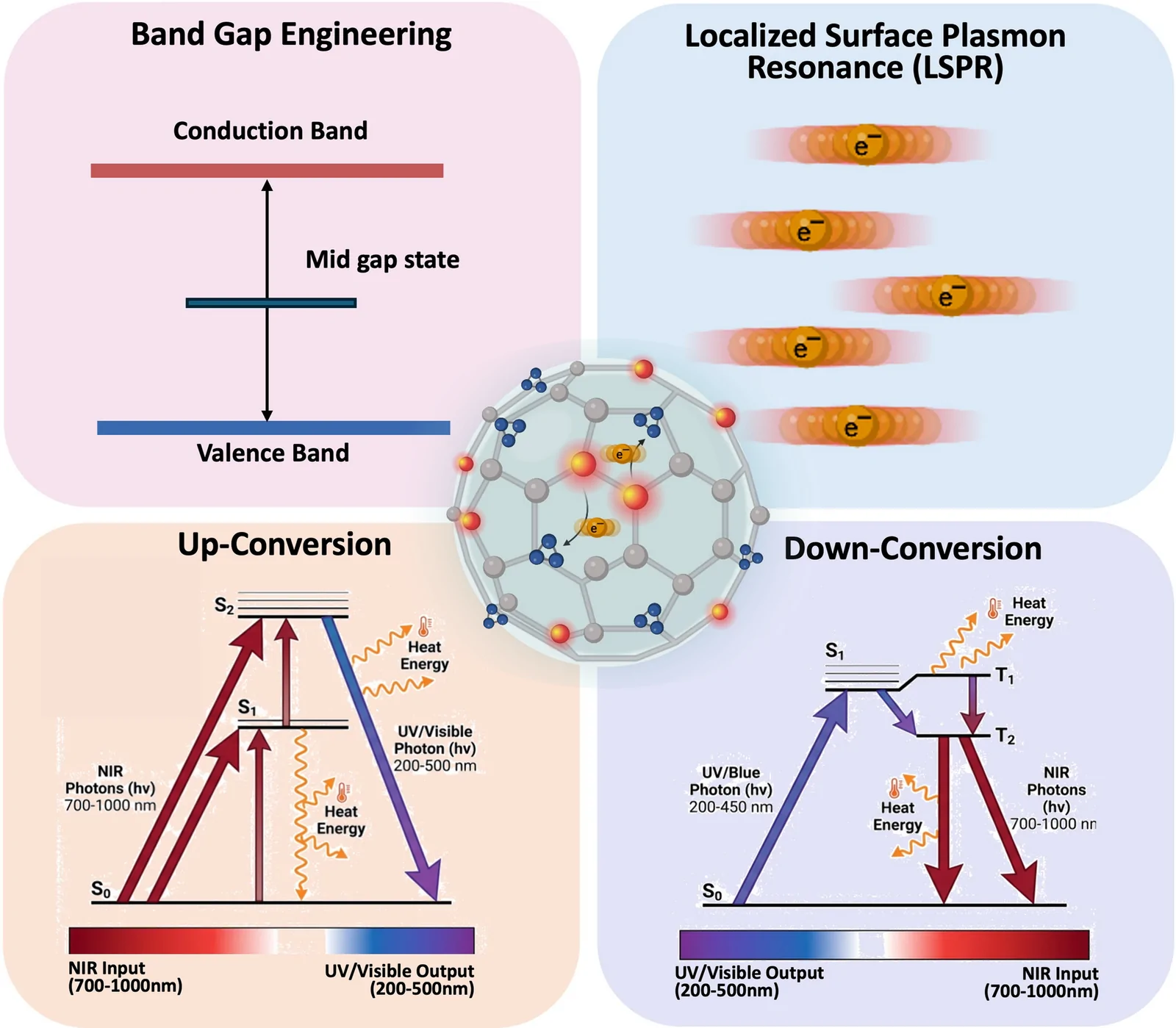
Modes of light harvesting domain: **A** Bad gap engineering done by the formation of mid gap state between conduction and valance band; **B** LSPR effect create by the simultaneous oscillation of high energy charge carriers; **C** Up-conversion chromophore formed by the conversion of low energy photons to high energy photons; **D** Down-conversion chromophore is represented by the transformation of high energy photons into low energy photons

The light harvesting domain of photonanozymes (PNZ) is an initiator of enzyme mimetic activity via its nano-scale architecture components. It captures high energy excited photons/hot carriers, filters and directs them towards the active site for catalysis. But the key feature of this domain is the selection of a portion of the solar spectrum which can efficiently transfer excitation energy and utilize the local electromagnetic field according to the molecular framework of photonanozymes. This holds the utmost importance because most of the times catalytic performances of PNZ are restricted by the mismanaged flow of photons more than their internal chemical kinetics. Light harvesting domains/complexes exist in following variable forms presented in Fig. 2 (C. Wang et al. 2021b; Zhang and Liu 2020).

#### Semiconductor band-gap engineering

Semiconductor band-gap engineering is one of the fundamental ways to design a light harvesting domain. It is a commonly acquired strategy for LHDs in nanostructures composed of semiconductors, as their band gaps can be adjusted by means of size quantization, doping, alloying, defect creation, dimensional confinement or heterojunction formation. Their solar spectrum absorption profile can be synchronized while preserving the catalytic redox potential. Semiconducting nanocrystals are mainly formed from metal oxides, quantum sized carbon materials, semiconducting heterostructures. The TiO₂ nanosheets absorb only 5% of the solar spectrum with the band gap of 3.2 eV. Nitrogen doping to these nanosheets narrows down the band gap to 2.4 eV while keeping the redox potential of nanosheets intact for peroxidase like activity (•OH generation) shown in Fig. 2A (Ali et al. 2025; Bai et al. 2018; B. Wang et al. 2021a).

#### Plasmon antennas and hot-carrier injection

Plasmonic antenna serve like an analogue of a radio antenna at the nano scale which captures high energy incoming photons, concentrates, packs their energy into a small package and transfers it to the catalytic site. The light harvest in plasmonic antennas is initiated by light irradiation at nanostructured noble metals like Au, Ag, Cu; their oxides, nitrides and chalcogenides. The high energy charge carriers in light strike onto the conduction electrons, energy is transferred and the electron cloud starts oscillating coherently which is called localized surface plasmon resonance (LSPR) present in Fig. 2B. Collective movement of electrons may lead to the formation of an extremely small bright, high energy, electromagnetic hot spots on the surface. These hot spots intensify the local electromagnetic field which is 10⁷ times stronger than the solar flux making the metal surface extremely hot and bright for microseconds (Guo et al. 2024; Adleman et al. 2009; Fasciani et al. 2011; Linic et al. 2015). This LSPR is short lived, heats up nearby molecules and boosts up the chemical reaction within microseconds. They transfer the hot electrons to the catalytic domain within femtoseconds and leave hot holes behind. The mechanism of LSPR is mainly dependent on the absorbed light, size and shape of nanostructures. By altering the size and shape of nanostructures, the spectrum of absorbed light can be easily adjusted and the energy is transferred via LSPR (Brongersma et al. 2015; Sundararaman et al. 2014). This mechanism of plasmonic antennas has wide applications in medical imaging. It has been demonstrated that the peroxidase-like activity demonstrated at 660 nm irradiation by core–shell Au@TiO₂ nanorods is 12 folds higher than TiO₂ (Lin et al. 2022).

#### Up-conversion and down-conversion chromophores

Chromophores absorb and reflect light irradiation according to their molecular arrangements. This potential of chromophores has been utilized to formulate the light harvesting domain of photonanozymes to stimulate enzyme like catalysis. Chromophores used in photonanozymes are usually optical frequency transformers engineered at the nanoscale which convert the frequency of light irradiation from one to the other by altering their energy while, keeping the number of photons same. In up-conversion, a chromophore captures low energy photons (near infrared spectrum) and converts them into high energy photons (UV or visible spectrum) Fig. 2C. Whereas a down conversion chromophore is an optical down shifter which absorbs a high energy photon (UV or blue spectrum) and splits it into two low energy photons (near infrared spectrum) Fig. 2D. (Bartusik-Aebisher et al. 2022; Eraky et al. 2025; Weingarten et al. 2017; Zhu et al. 2020) During this conversion cascade, energy is generated in the form of heat. The spectral shift increases the potential of photonanozymes to initiate and propagate catalysis on those wavelengths which were not operable earlier. Up-conversion has been demonstrated in β-NaYF₄:Yb^3^⁺,Er^3^⁺ nanocrystals which sequentially absorb two or more photons from the near infra range (980 nm). In these crystals,energy is transferred to Er^3^⁺ by Yb^3^⁺, hence, emitting one photon from the green range (540 nm). This up converted photon excites the TiO₂ shell (requires ultraviolet for activation) catalyzing the decomposition of H₂O₂ (Wang et al. 2011).

Semiconductor band-gap engineering offers the highest quantum yields among the three domains, with recent reports achieving a apparent quantum yield (AQY) of 1.5%—35.6% at the UV to visible light spectrum for optimized heterojunctions. However, this approach suffers from rapid electron–hole recombination and limited absorption in the visible spectrum (Rao et al. 2025). Plasmonic antennas and hot-carrier injection provide tunable absorption across visible to NIR regions, yet their practical utility is severely constrained by extremely low quantum efficiencies; a recorded high AQY of 0.38% at 800 nm has been reported for Pt-tipped Au nanorods with hot carrier utilization often being < 1% of absorbed energy. Plasmon decay produces hot carriers through Landau damping, but the efficiency of hot-electron photoemission remains very low (Hu et al. 2023). Up-conversion and down-conversion chromophores enable catalysis at previously inaccessible wavelengths by converting NIR to UV/visible light, facilitating deep-tissue applications. However, up-conversion systems exhibit inherently low quantum yields, limiting their catalytic turnover (Peng et al. 2025; Zhao et al. 2025) as mentioned in Table 1. A fundamental dilemma has been found: materials with higher quantum yield and stability often lack optimal catalytic turnover and vice versa. Hence no single domain outperforms, the optimal choice depends on the optimization of quantum yield, catalytic turnover and stability for specific applications. The yield of down-conversion materials are usually measured in Photoluminescence Quantum Yield (PLQY) indicating numbers of photons emitted per number of photons absorbed. The PLQY can exceed 100% which means one absorbed photon produce two emitted photons in terms of efficiency. Hence, down conversion is measure of emission of energy/photons rather than chemical catalysis (Liang et al. 2026).

**Table 1:** Characteristics comparison of different light harvesting domains

Parameter	Light Absorption Range	Apparent Quantum Yield (AQY)	Turnover frequency	Reusability/stability	References
	(nm)	(%)	(mmol h^− 1^ g^− 1^)	(Cycles/hour)	
Semiconductor Band-Gap Engineering	100–700	1.5 – 35.6%	0.0137- 121.01*	2–30/8–32	(Rao et al. 2025)
Plasmon Antennas & Hot-Carrier Injection	400–900	0.38%	0.55	10/3	(Hu et al. 2023)
Up-Conversion (UC)	100–700	1.8%	0.00407^*^	4/32	(Peng et al. 2025; Li et al. 2025; Weitzel et al. 2025)

^*^Unit is standardized to mmol h^−1^ g^−1^

Semiconductor engineering suits high-efficiency photocatalytic degradation and energy conversion where UV/visible light is available, offering high quantum yields and tunable band gaps through doping, heterojunction formation, and defect engineering. Recent advances demonstrate that optimized semiconductor heterojunctions can achieve apparent quantum efficiencies exceeding 60% for solar fuel production, while modified TiO₂ systems maintain robust performance for water treatment applications under UV/visible irradiation (Kim et al. 2019; Takata et al. 2020; Wang et al. 2014). In contrast, plasmonic antennas are ideal for biosensing and imaging applications where wavelength tunability and local field enhancement are critical. Their localized surface plasmon resonance (LSPR) enables absorption tuning across visible to near-infrared regions by adjusting nanoparticle size and shape. This generates an electromagnetic field which is stronger than incident light, making them highly sensitive platforms for biosensing and photoacoustic imaging (Kim et al. 2019). Up-conversion chromophores are uniquely suited for biomedical applications requiring deep-tissue penetration, such as photodynamic therapy and in vivo bioimaging, where NIR excitation enables therapy at depth while minimizing damage to healthy tissues. By converting low-energy NIR photons (980 nm) into high-energy UV/visible emissions, lanthanide-doped upconversion nanoparticles achieve tissue penetration depths of several centimeters, enabling site-specific reactive oxygen species generation and real-time tumor imaging with minimal phototoxicity to surrounding healthy tissues (Wang et al. 2026a, b; Zhou et al. 2026a, b).

Thus, the selection of an appropriate light-harvesting domain is dictated by the specific application requirements, whether high quantum efficiency for energy conversion, field enhancement for biosensing, or deep-tissue penetration for therapeutic interventions with each domain offering distinct advantages that complement the others in the photonanozyme platform. The light-harvesting domain thus initiates the catalytic cascade, but the ensuing photochemical energy must be precisely channeled to the enzyme-mimetic active site to achieve the desired substrate turnover and reaction selectivity.

### Enzyme-mimetic active site

The enzyme mimetic active site of photonanozymes (PNZ) is designed in a way that mimics the active site of an enzyme at the nanoscale. It is a three-dimensional structure which contains residues for substrate binding and catalysis, activated by excitons, catalyzing the formation and breakage of bonds. These active sites can be hybrids (between natural biomolecules and synthetic compounds) or engineered completely. They are formulated and engineered in a way to contain geometric pockets, or electronic defects or single atoms. The turnover frequency, selectivity and regulation are mainly modulated by wavelength, intensity and pulse illumination (Y. Liu et al. 2021c; C. Wang et al. 2021b; Zhang and Liu 2020). Their dynamic nature under light irradiation reconfigures the photonanozymes continuously, hence regulating their catalysis (Gong et al. 2023). Natural photoenzymes are highly specific because of their substrate binding domain which mechanistically alters the substrate by the help of photons of the specific light spectrum. Synthetic photonanozymes follow the same architecture by the introduction of defects over their surface which are characterized by atomic dislocations, absence, vacancies or structural anomalies. They directly influence the chemical, physical and catalytic properties of photonanozymes. Regulating these defects can alter their functionality and eventually their applications (C. Wang et al. 2021b; Yang et al. 2021). Some of the hallmarks that determine the mechanistic consequences of photonanozymes are mentioned as follows.

#### Electronic states and charge trapping

The active site must have the ability to temporarily store the electrons or holes generated by the irradiation of light at the harvesting domain in order to perform the catalysis. The conversion of kinetic energy of photons to chemical energy is mediated by the electronic state of the active site of photonanozymes. The high performance efficiency of the catalytic system is primarily based on the electronic state of the active domain which traps, stores and shuttles the charge carrier in a temporal manner (fastest reaction rate is considered the best). The catalytic reaction requires multiple electrons. Therefore, the active site must hold the charge carriers long enough to utilize them in the specified reactions. The active site must have the energy level that overlaps the energy required to carry out the redox reaction at the substrate. Moreover, it also spatially separates the substrate to prevent the recombination of the electrons and holes (Chen et al. 2017; Xu et al. 2024). The charge trapping by the active site is mediated by mid-gap defects, single atom d-orbitals, delocalized π* bands or photothermal charge storage. Mid-gap defects like cyano defects (-C≡N) are characterized by the presence of an energy level at the mid-point between the conduction band and the valence band which stores electrons and helps conduct the redox cycling. In some of the dopants, the d-orbitals are usually split into their sub-state orbitals, i.e., t_2g_ and e_g_ (for octahedral) and e and t_2_ (for tetrahedral) located above the Fermi level which is suitable for redox reaction. Delocalized π* bands trap and store the electron by introducing π orbital below (0.7–0.9 eV) the conduction band minimum (CBM). This is done to prevent the recombination of electrons and holes before their death (Goh et al. 2017; Kong et al. 2025; Liu et al. 2019). During photothermal charge storage, high energy vibrating nanoparticles activated by the photothermal energy store the hot electrons on the surface layers (within nanometers) and inject them into the adjacent metal within nanoseconds which conducts the redox reaction.

#### Micro-environment and hydrophobic gating

**Fig. 3:**
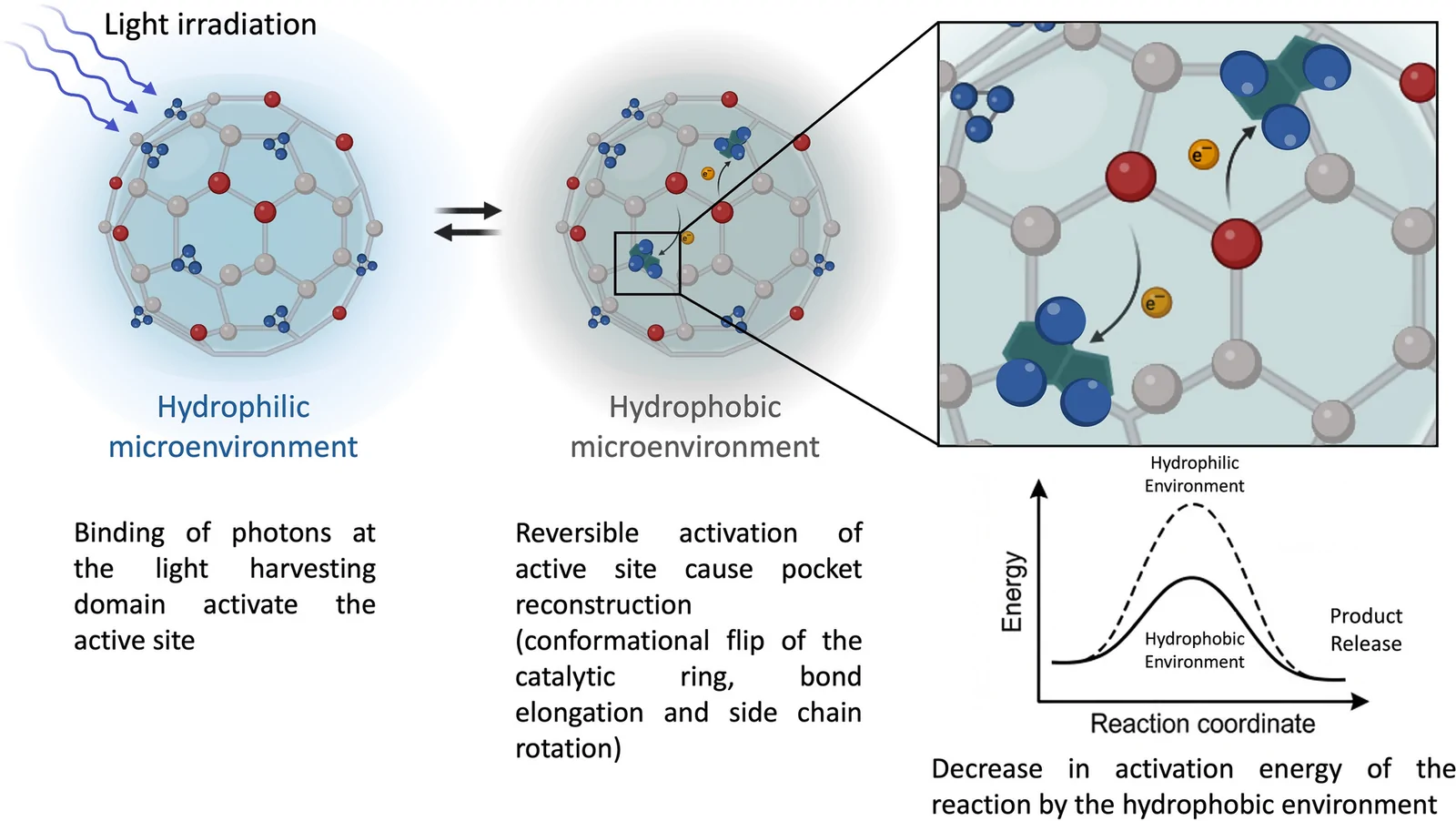
Hydrophobic gating of enzyme mimetic active site: Reversible conversion of hydrophilic microenvironment to hydrophobic microenvironment which reduce the reaction activation energy of photonanozymes

The active sites of enzymes frequently provide and maintain a hydrophobic microenvironment which eliminates water and reduces the activation energy of a reaction. It is the minimum amount of energy required to make or break the bond. The reduced activation energy means less amount of energy required for the given purpose which makes the reaction easy to be conducted. Photonanozymes reduce the activation energy by inducing the reversible hydrophobicity at the active site (surface defects or single atom sites) as depicted in Fig. 3. Mostly this can be achieved by doping the photonanozymes with fluorine or alkyl-silane. These dopants exclude water by altering the contact angle between active site and substrate, binding of super-hydrophobic co-catalyst along with substrate, forming a hydrophobic layer around the photonanozyme, or generating the tunable enantioselectivity for the hydrophobic compounds (Biler et al. 2020; Blaha-Nelson et al. 2017; Deng et al. 2025; Paul et al. 2024; Tang et al. 2007). Carbon nitride based nanomaterials contain a single iron atom (Fe–N-C) as a catalytic site for peroxidase like activity. Its surface is functionalized with fluoroalkyl silanes (hydrophobic gates for active sites) which reduce the activation energy up to 0.3 eV and speed up the reaction. The hydrophobic layer eliminates the water molecules from the active site as it competes with the substrate (H₂O₂) affinity for the catalytic site. Moreover, water molecules also stabilize the reaction intermediates which slows down the reaction rate. Hence, hydrophobic gating serves as a filter which selectively facilitates the entry of substrate and repels the water molecules (Ji et al. 2021).

#### Substrate-induced dynamic restructuring

Substrate induced dynamic restructuring is one of the crucial phenomena used by enzymes to ensure the perfect and maximum fit of the substrate at the active site. Binding of the substrate at the active site alters the conformation of catalytic residues. Conformational changes include bond elongation, side chain rotation and pocket constriction. In photonanozymes, this phenomenon is mediated by the attachment of external stimuli like photons which induce real time and reversible change in the atomic rearrangement at the active site. The static residues at the active site optimize their arrangement to make the maximum contact with the substrate molecules, converting it into a living catalyst. This lowers the activation energy, hence making the reaction faster. After the completion of the reaction, the active site attains its original conformation (Jiang et al. 2019; Yu et al. 2023; Zhang et al. 2024). Coupling it with surface defects boosts substrate selectivity, self-healing with enhanced product yield.

This phenomenon has been demonstrated in short lived peroxo bridge (M–O–O–H or M–O–O–M) formed by the metal sites present on the oxide or metal nitride surface of PNZs. They are formed when unsaturated exposed metal atoms embedded on the solid surface adsorb oxygen containing molecules. The metal atoms donate their lone pair of electrons to oxygen containing molecules, hence activating the oxygen. A peroxo-bridge is formed between the activated oxygen and the metal atom (M–O–O–H or M–O–O–M), which easily transfers oxygen atoms or electrons to the next step of the catalytic reaction. Formation, structure and reactive potential facilitate photo-oxidation or oxygen transfer reactions in photonanozymes (Zhang et al. 2020a, b).

#### Multi-functional single-atom sites

Single atom sites or single atom catalysts are like shrinking macroscopic metals into a single atom while preserving all the properties and function of that metal. They are made by embedding lonely metal atoms on the surface of a host lattice usually made up of carbon, metal or non-metal oxides or metal organic frameworks. These tailored single atoms possess all the potential to perform catalysis which is achieved by a three in one consequence, i.e., maximum atom utilization, minimum diffusion barriers and unique electronic states. Their maximum atomic utilization can be achieved by dispersing them throughout the host lattice and replacing the bulk atoms with low or no catalytic efficiency. They speed up the reaction rate due to minimum interatom diffusion barriers as all the atoms are dispersed around the lattice and the reactant can reach the catalytic atoms instantly. Single atom sites have a unique electronic state. They split their empty d-orbitals into "e" and "t" sub-orbitals with a mid-gap state that thrives for electrons. For nanoparticles having octahedral geometry, the sub d-orbitals are t_2g_ and e_g_, while tetrahedral geometry is characterized by e and t_2_ sub-orbitals. As the photons from the irradiated light strike, electrons are directly transferred from the orbitals of the support ligand (embedded over the lattice) present in the vicinity of single atoms. This is known as ligand to metal charge transfer (LMCT) (Huang et al. 2023; Liu et al. 2025; Shi et al. 2023; Zhai et al. 2020). This regulates the electron density within the single atoms which selectively hold the reactant and convert it into the respective product. This has been observed in Rh single atom nanozymes which demonstrate the ascorbate peroxidase like activity for detection of ascorbic acid by electrocatalysis. This single atom based biosensor operates at very low voltage and shows excellent performance for the detection of ascorbic acid in human subjects and nutritional products (Shi et al. 2023).

#### Regulation by external stimuli

Photonic gating acts as allosteric effectors for PNZs producing spatiotemporal responses. Like allosteric effectors, photons bind at the pocket away from the active site. They induce conformational changes at the active site and regulate the catalysis. The mechanism of photonic gating works by switching "on" and "off" the material by the light stimulus. Light irradiation at the allosteric site induces the structural change at the active site. Mostly, the structural changes exist in three forms which are conformational flip from trans to cis or cis to trans orientation, opening or closing of the catalytic ring, folding or unfolding of β-sheets. These structural changes alter the pocket volume which either induces or restricts the activity of photonanozymes. These catalytic responses are generated within sub-milliseconds (Chen et al. 2017; Neri et al. 2017). Azobenzene switches on the peroxidase activity by flipping the conformation from trans to cis when ultraviolet light of 365 nm is irradiated and reverts back to trans conformation by the visible light irradiation. (Zhu and Zhou 2018). Diarylethene switches on by the striking of 520 nm green light which opens the ring and catalyzes the CO₂ reduction (Majee and Presolski 2021). While light oxygen voltage sensing domain 2 (LOV2) is a protein complex made of 5 β-sheets flanked by α-helices. The LOV2 domain is responsive to blue light and switches on the cleavage of DNA fragment as the compatible spectrum of light strikes (Wu et al. 2009). These catalytic responses are reverted to their original form when light is switched off. The external stimulus of photonic gating is the key component to induce the spatiotemporal control, reversible cycling with efficient utilization of energy in the PNZs.

These hallmarks represent a sophisticated biomimetic paradigm but offer practical limitations. Electronic states designed to trap charge represent the compromised storage capacity leading to their precatalytic recombination. Hydrophobic gating of the surrounding environment is effective in reducing the activation energy of the reaction but complicates the reaction engineering of hydrophilic substrates. Substrate induced dynamic restructuring generates irreversible change by the extended exposure to irradiation. Substrate-induced dynamic restructuring is frequently irreversible under prolonged irradiation, reducing catalytic stability. Single atom sites are efficient but form clumps by the usage over the time. Moreover, photo-gated photonanozymes suffer slow kinetics with photodegradation. They individually and collectively restrict the catalytic stability with compromised recyclability of photonanozymes. These challenges emphasize the need for modular design strategies with applicable and practical comprehensive stability assessments.

The enzyme-mimetic active site executes the primary catalytic chemistry, yet its performance is ultimately dictated by the nano-environment, the nanoscale reaction chamber that spatially confines intermediates, steers substrate orientation, and mediates charge-carrier dynamics.

### Nano-environment

The microenvironment created within and around the PNZs theoretically plays the role of a "nanoreactor" having nanoscale size surroundings containing walls, pockets and interfacial fields. This microenvironment of PNZs is also an integral part of their activity and is considered as one of the components of PNZs. It organizes the conformation of the substrate, detaches the redox half reactions from each other and prevents the recombination of electrons and holes without influencing the additives of the bulk phase. The mechanistic approaches and design schemes which modulate the catalytic output are mentioned in the upcoming section.

#### Geometric confinement and substrate pre-organization

**Fig. 4:**
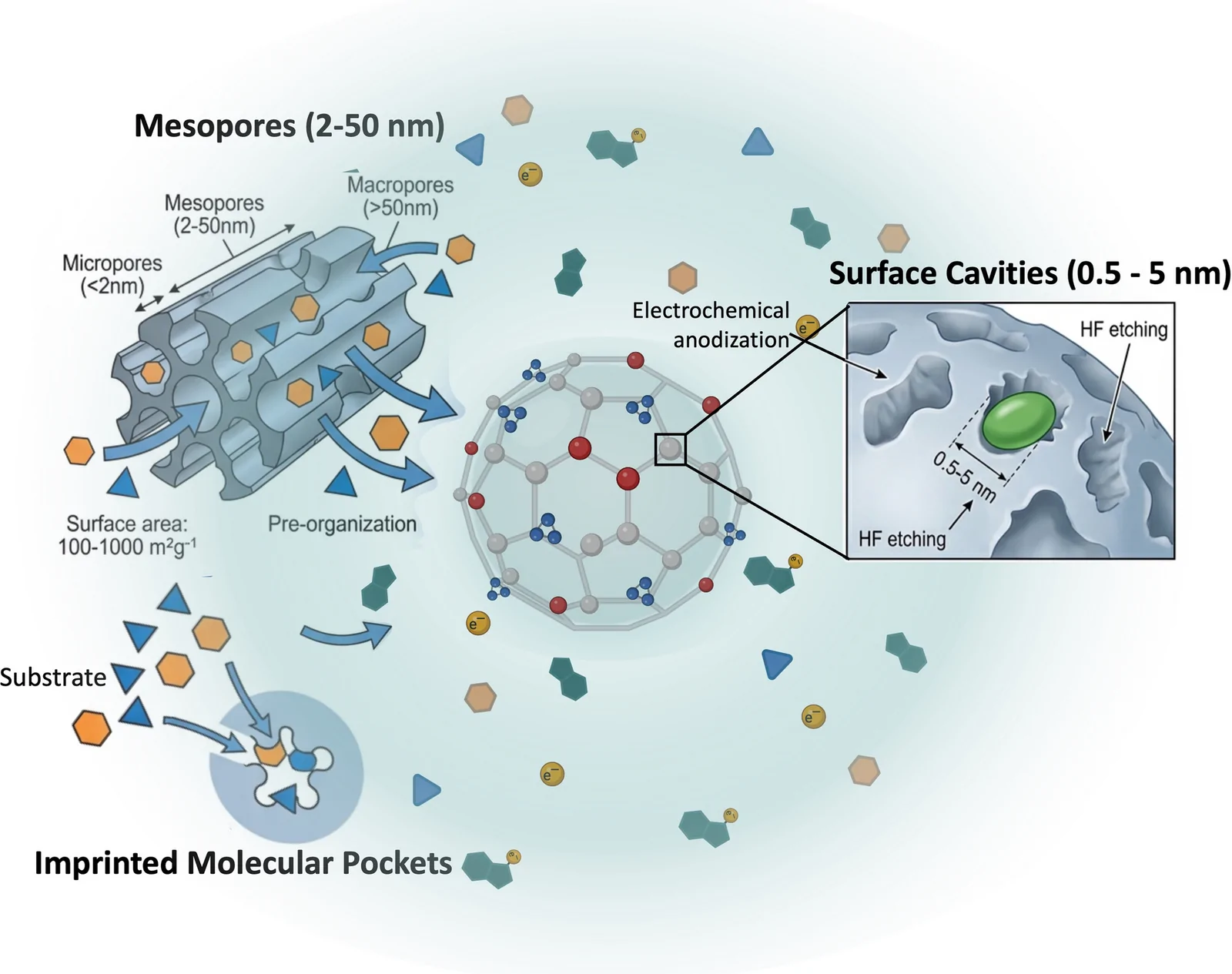
Nano-environment in catalysis: Tailoring nano-environment around the photonanozymes to induce desired geometric confinements which pre-organize substrate for efficient catalysis

Geometric confinement and substrate pre-organization are key players for efficient catalysis. In natural enzymes, geometric confinement is characterized by hydrogen bonded networks, hydrophobic grooves and π–π stacking which help in organizing the substrates at the active site before catalysis (Adak et al. 2022). Artificial engineered PNZs follow this architecture of geometric confinement and substrate pre-organization via forming mesopores, surface cavities and imprinted molecular pockets. These are proportionate with the kinetic dimensions of the substrate molecules, hence lowering the activation energy and enhancing the turnover frequency of the reaction exhibited by Fig. 4 (Deogratias et al. 2022; Wordsworth et al. 2020).

Mesopores are small passages of 2–50 nm bridging micro and macro pores offering enhanced surface area to adjust the active site to efficiently diffuse the reactant. They are formed by soft and hard templating, sol–gel method and blocking copolymer self-assembly. Soft templating generates flexible tunnels whereas, hard templating provides hard frameworks. Washing-off soft template leaves a pore that replicates its architecture. However, maintaining the structural integrity during the template removal is a critical challenge because the pores collapse or replicate incompletely which often compromises the catalytic performance, requiring the defined control over synthesis conditions for optimal functionality (Yalin He et al. 2025b; Zahoor et al. 2026).

Surface cavities are nanosized depressions formed over the structure of photonanozymes of 0.5 to 5 nm through electrochemical anodization or hydrofluoric acid etching (HF etching). In anodization, electric current is generated in the acid bath to produce a porous layer of oxides. While HF etching is used to carve cavities by the chemical dissolution method. With widespread applications and benefits, this method caters to a critical challenge of accuracy in pore uniformity and prevention of structural collapse during synthesis. The balance of oxide formation and acid dissolution is highly sensitive to voltage, temperature and etching time. Reproducibility is a point of critical concern because minor variations in parameters remarkably vary the pore morphology with catalytic efficiency. Nevertheless, these cavities enhance substrate confinement, accelerating diffusion and lowering activation energy for improved catalysis (Zhan et al. 2024; Zhong et al. 2024).

Imprinted molecular pockets are nanoscale pockets etched around the template (substrate, ions or dyes). As the material polymerizes around the template, it is removed chemically forming a complementary structure. All the engineering approaches have their individual significances in geometric confinement and substrate pre-organization. Mesopores distill the reactants by shortening the diffusion path. Surface cavities tightly hold the substrate by van der Waals forces and π–π stacking. Imprinted pockets follow the induced-fit model of enzyme substrate interaction by promoting the shape specific catalysis, photoswitchability and enantioselectivity of the substrate molecules (Bu et al. 2024; Zhou Zhang et al. 2025b; Zhou et al. 2026b).

#### Micro-environment polarity and hydrophobic gating

The polarity of the nano environment surrounding the photonanozymes critically regulates their performance by controlling the electron transfer kinetics. Polar environment (e.g., water, ε ≈ 78) binds the charges with high affinity and increases the reorganization energy (λ) with HOMO–LUMO gap. This requires higher driving force (ΔG) for ET and eventually decelerates the reaction rate. Whereas non-polar hydrophobic nano environment (ε ≈ 2–8) considerably reduces the λ and ΔG which accelerates ET up to 10 folds according to the Marcus theory. (Jensen et al. 2025; Kuang et al. 2021; Liu et al. 2023; Marcus 1956; Xiunan et al. 2022).

For instance, fluorinated TiO₂ nanosheets demonstrate reduced λ (0.34 eV) with three times faster peroxidase like activity compared to their hydrophilic counterparts (λ = 0.68 eV). Though hydrophobic gating improves the ET, substrate compatibility is a hurdle created by the non-polar environment to refrain from the attachment of hydrophilic substrates. Hence, polarity must be adjusted to the substrate's composition which balances the optimal ET kinetics with effective substrate pre-organization. This tuning of the nano environment copies the solvation shell of natural enzymes, positioning hydrophobic gating as a strong design lever for achieving high efficiency, programmable catalysis in photonanozymes (Ji et al. 2025; Yu et al. 2021).

#### Defect-mediated charge separation

Defect mediated charge separation is a space and time bound series of reactions occurring inside the nano-environment of photonanozymes. The atomic size defects are engineered on the surfaces of photonanozymes which act as a quantum cavity and perform the charge separation and catalysis within microseconds because of their ultra-small size. The external space of 0.5–2 nm surrounding the catalytic site is a dynamic regulation dial that is mediated by defect density, lattice polarity and mechanical strain from the surface conformation (Bai et al. 2018). These mediators are altered by electronic band edge engineering, electrostatic field reconfiguration and vibrational degree of freedom respectively. However, the dynamic modulation of the microenvironment is performed by means of ionic strength, temperature, solvent concentration, pH, polarity and structural geometry (Maarisetty and Baral 2020). Defect density controls charge separation through the intentionally created mid-gap state (oxygen vacancies, interstitials) which trap photogenerated charge carriers. Defect population is modulated by ionic strength, oxygen availability and synthesis temperature. Higher ionic strength creates more vacancies, while oxygen-rich environments shift defect levels toward the conduction band, weakening trapping efficiency. Lowering the conduction band edge (CBE) by 100–200 meV enables deeper, longer charge trapping which enhances separation and catalytic activity. However, disproportionate defects cause carrier recombination and reduce the efficiency. Hence, optimal engineering of defects to balance the trap density with electronic alignment must be incorporated for optimal photocatalytic performance (Muhammad et al. 2024; Pastor et al. 2022; Zhekun Zhang et al. 2025c).

Lattice polarity directs charge separation through dipole-like surface cavities (0.5 −2 nm) that channel electrons to the active site. This effect is regulated by altering the microenvironment, i.e., decreasing pH protonates the hydroxyl group which doubles the local negative field and intensifies the charge trapping cavities. Higher dielectric constant gained by introducing polar bonds increases electron attraction and improves separation. Contrarily, increasing salt concentration suppresses the Debye layer, shortens the charge pathways and reduces the energy loss. Hence, optimizing pH, dielectric environment, and ionic strength tunes the internal electric field which enhances the electron–hole separation without material modification. However, maintaining cavity stability under fluctuating conditions and over polarization induced recombination which pose critical challenges over functionality (Ali et al. 2025; Bai et al. 2018; Feng et al. 2024; Maarisetty and Baral 2020). These critical challenges of lattice polarity fluctuation can be stabilized by incorporating heterostructure frameworks that provide a uniform charge-transport network, ensuring real-time perturbation of interfacial carrier density and suppressing potential drift. Over-polarization induced recombination can be mitigated by interfacial charge redistribution which lowers the reaction energy barrier and facilitates electron transfer while preventing excessive carrier accumulation.

Mechanical strain governs the charge separation by means of vibronic coupling controlled by phonons. Low-frequency (soft) phonons facilitate electron trapping in deep defect states, while high-frequency (stiff) phonons provide the energy necessary for electron release to catalytic sites. External compression lowers phonon energy, prolonging charge confinement. However, excessive strain risks lattice distortion, and precise phonon engineering remains synthetically challenging for reliable photocatalysis. Precision in strain engineering by regulated lattice mismatch in heterostructures or dopant induced stress can overcome these challenges without compromising the structural integrity (Stroscio and Dutta 2001; Chen et al. 2015; Mao et al. 2025; Tong et al. 2020).

#### Microenvironment feedback loop

The dynamic association of these three features of the microenvironment transforms the flexible surface defects of PNZs into a live and self-tuned quantum cavity that undergoes a feedback mechanism. The density of the defect is directly affected by the surrounding pH. Increase in pH (H + ions concentration), generate more oxygen vacancies and more intense cavities which trap the electrons longer. Thus, preventing the recombination with the holes (N. Zhao et al. 2021b). Simultaneously, escalated salt concentration squeezes the electric field which shallows down the charge funnel resulting in the rapid pushing of electrons towards the catalytic site (Clavier et al. 2016). Thermal treatment strains the lattice which stiffens the phonon vibrations, hence electrons are transferred quickly to initiate the reaction (Tong et al. 2020). The optimum conditions result in their dynamic combinatorial effects within milliseconds which prevent the electrons and holes from recombining and swiftly transfer them to the reaction to initiate the catalysis.

#### Heterojunction interfaces and Z-scheme charge highways

When electrons get excited, the excited electrons are shifted to conduction band minima (CBM) leaving holes in the valence band maxima (VBM). Their directional flow may lead to their recombination which leads to their elimination and energy release in the form of heat or light. The charge carrier recombination can be avoided by the heterojunction and their associated mechanisms. Heterojunctions are created when two different semiconductors are joined, to increase the efficiency of catalysis and to prevent the recombination of charge carriers in photonanozymes. Both semiconductors have variable electronic band structure which places their VBM and CBM at different positions. This gives a straddling (zigzag) alignment to the collective bands ofthe semiconductor. At the interface of both semiconductors, internal electric field is created which directs the flow of charge carriers. This is one of the basic and strong attributes of photonanozymes mediated catalysis. (Schumacher and Marschall 2022; Sudrajat and Nobatova 2025). The mechanism of action is divided into four interconnected steps.

Band Engineering at Atomic Interface: The VBM and CBM of semiconductors (forming a zigzag pattern) are connected at the heterojunction. Energy levels (specifically Fermi level) are equilibrated by bending their VBM and CBM. The Fermi level is the ground state of an electron in which it contains the highest energy. The bending will create a built-in electric field which has a stronger energy magnitude than the external field and facilitates the efficient carrier separation (Balapure et al. 2024; Sudrajat and Nobatova 2025).

Formation of Internal Electric Field: Different materials have their Fermi level located at different regions of the electronic band structure. The Fermi level of conductors is present in the CBM, non-conductors in the VBM and semiconductors in the band gap. The n-type electron rich semiconductors are found near the CBM edge and p-type proton rich semiconductors near the VBM edge. To adjust the Fermi level of heteroatoms, the electron rich bands are bent downwards while, the proton rich bands are bent upwards directing the electrons downhill and holes uphill keeping them separated from each other. Because of bending, the bands of fixed polarization of charges are formed in which the positive band of one atom faces the negative band of another atom. Due to opposite poles both hold each other at the particular distance and create an internal electric field (Sudrajat and Nobatova 2025; Vinh et al. 2025).

Directional Charge Flow and Suppression of Recombination: The staggered alignment of the energy bands helps electrons to move from one semiconductor to another semiconductor of a heteroatom. The electron moves from (lower energy atom to high energy atom) lower valence band to lower conduction band then jumps to higher valence band to higher conduction band. Forming a pathway which resembles the letter "Z" therefore, this is called as "Z-scheme charge highways". The spatial separation will prevent the recombination of charge carriers which extends their lifetime and enhances the turnover frequency (Dong Liu 2021; Schumacher and Marschall 2022).

Tunability by Mechanical Strain and External Factors: The generated internal electric field of photonanozymes forms a slope which pushes electrons and holes in different directions. External strain regulates the steepness and slant of the electric field, which varies according to the material used. It is in the form of electric, magnetic or their combination. Electrically the internal field is regulated by means of band bending control and Debye Layer compression. In band bending control, external field bends the bands which slant the energy level even more and electrons move downwards, and holes move upwards (Gao et al. 2019; Sudrajat and Nobatova 2025). In Debye layer compression, high salt concentration layer squeezes the electron clouds and reduces the size of the electron funnel which prevents them from recombining with the holes. Applying 0.1 V to g-C₃N₄/MoSi₂N₄ nanoparticles transfers 73% of electrons in 223 femtoseconds. Applied magnetic strain prevents the recombination by applying Lorentz force which moves electrons and holes in opposite directions or generating spin polarized bands in which energy bands allow unidirectional spin (Wang et al. 2025). Synergistic approach encompassing both electric and magnetic field has been observed in TiO₂ nanowires in which – 0.6 V with ultraviolet irradiation inhibits the recombination (Pesci et al. 2013).

#### Practical impact on catalysis

A heteroatom of TiO₂/BiVO₄ was formed in which internal electric field formed at the junction of both atoms directs the electrons to TiO₂. This heteroatom performs water splitting and organic degradation mediated by light stimulation (Wang et al. 2016). The heterojunction of g-C₃N₄/WO₃ possesses efficient redox potential with selective partial oxidation of organic compounds with > 95% product formation rate (Li and Wang 2024).

The practical influence of heterojunction in separating the charges is however compromised by the interfacial defects, ambiguity of Z scheme and realistic translation of ultrafast dynamics from idealized conditions to realistic porous architectures. This can be resolved by tuning the external field which appears promising but operational complexity and scalability pose a threat to their implementation. Thus, integration of heterojunction requires delicate coupling of defect engineering with reactor design.

#### Persulfate and radical-scavenging nano-environments

Persulfate molecules are the electron scavengers present in the nano environment that prevent the charge recombination. They accelerate the reaction process by trapping photogenerated electrons to produce SO₄•⁻ radicals while holes oxidize the pollutants. This dual functionality of persulfate molecules enhances the degradation efficiency and supports catalytic cycles through persulfate regeneration. The Fe₃O₄-ZnIn₂S₄ heterojunction demonstrates this mechanism, achieving complete degradation of sulfadiazine via •OH and SO₄•⁻ generation. However, challenges remain, including the potential for excessive radical quenching, persulfate consumption leading to secondary contamination, and pH-dependent performance, necessitating careful optimization for practical applications in wastewater treatment.

These potential challenges can be eliminated by maintaining the equilibrium between radical and non-radical pathways. This is achieved by pH control, adjustment of operational parameters and catalyst modification. Optimizing reaction pH and incorporation of chelating agents prevent the excessive radical quenching and enhance the efficient utilization of persulfate radicals (Aali et al. 2025; Gao et al. 2019; Guo et al. 2022; Tian et al. 2022; Wang et al. 2026a, b).

The three-domain biomimetic design strategy of photonanozymes confronts fundamental limitations that restrict its catalytic potential. Interfacial defects between domains critically hinder their performance. Uncontrolled or unprogrammed defects resist the charge transfer obstructing electron mobility and eventually catalytic turnover (Guan et al. 2025; Tripathy and Baral 2025). Light harvesting faces a functional restriction, as the light absorption is improved, electron–hole recombination is enhanced hence, wasting the absorbed energy in the form of heat (Li et al. 2026; Zhang et al. 2016). Practical challenges limiting the efficiency, selectivity and broader applications are under-explored catalytic mechanisms, ambiguous structure–activity relationships with dependence on non-specific surface sites (Sharma et al. 2026; Xu and Fan 2026). The synergistic, competitive, and antagonistic interactions of multi-enzyme-like activities result in complex, nonlinear behavior which makes their performance hard to predict (Jiang et al. 2024). While conventional nanozymes still underperform natural enzymes in catalytic activity and offer limited precision in functional design (Sharma et al. 2026). The dependence on empirical design paradigms and first-principles calculations, which are computationally expensive, further hinders theoretical predictions (Chen et al. 2023a, b). These limitations including interfacial defects, trade-offs between light absorption and charge recombination, ambiguous mechanisms, and reliance on trial-and-error methodologies underscore that the three-domain framework demands fundamental rethinking, requiring advances in interfacial engineering, precise defect control, and strategies to decouple light absorption from charge recombination to achieve practical applicability.

Collectively, these approaches present a well-organized regulation of photonanozymes in terms of polarity of surface, defect chemistry, molecular conformation of pores and interfacial kinetics that arrange substrate molecules for maximum interaction. They also prevent the damaging recombination of electrons with holes, which maximizes the performance of photonanozymes for versatile enzyme mimicking catalysis characterized by their widened applications in diverse domains.

## Enzyme-like activities of photonanozymes

Photonanozymes are a complex bio-inspired system which imitates natural enzyme-like activity utilizing light energy as a catalyst. Contrary to natural enzymes which create and make active sites available via complicated tertiary structure formation; the catalysis (redox potential) of PNZs is mediated via their distinct electronic and molecular surface properties activated by light irradiation of a specific spectrum. They have been shown to perform the enzyme-like activity of different enzymes like peroxidase (POD), catalase (CAT), oxidase (OXD), superoxide dismutase (SOD), nitroreductase, DNA-cleaving nuclease and fatty-acid decarboxylase (FAP) (Wang et al. 2020; Wei and Wang 2013; Wu et al. 2019; Yang et al. 2021).

### Peroxidase-like (POD) activity

The PNZs perform the peroxidase-like enzyme activity which imitates the function of natural peroxidase enzymes like horseradish peroxidase. The POD activity is composed of the decomposition of hydrogen peroxide (H₂O₂), to produce the highly reactive oxygen species (ROS) hydroxyl radicals (•OH). The reaction is initiated by the generation of hot carriers by the produced surface plasmon resonance effect (SPR). The resonance decay produces electron–hole pairs which are transferred to the attached H₂O₂ or O₂. The hot electron is injected into the antibonding σ* orbital of H₂O₂, weakening the O–O bond and generating two •OH by homolytic cleavage. These •OH radicals can transfer electrons to metal ions and convert them into a reduced state (e.g. Fe^3^⁺ to Fe^2^⁺). Simultaneously, these hot holes oxidize the electron donors like 3,3′, 5,5′ tetramethylbenzidine dihydrochloride (TMB) which is a common peroxidase substrate. The light mediated excitation provides energy to overcome the activation barriers while the field created by thermal vibration increases the concentration of reactants at the surface which speeds up the reaction rate and activity as compared to inactivated nanozymes (Cao et al. 2024; Lin et al. 2022; Su et al. 2024; Ye et al. 2024). This has been observed in Au doped Pt-TiO₂ nanocomposites demonstrating enhanced peroxidase like activity by the oxidation of O-Phenylenediamine (OPD) under the ultrasound stimulus. The improved activity under ultrasound stimulus can be efficiently utilized in the therapeutic platform to generate reactive oxygen species (ROS) (Ameur et al. 2025).

### Oxidase (OXD) like activity

Photonanozymes perform oxidase (OXD) like activity induced by light irradiation to generate superoxide radicals (•O₂⁻) or other reactive oxygen species (ROS) produced by the reduction of molecular oxygen (O₂). The radicals eventually oxidize other substrates independent of H₂O₂. The key difference between POD and OXD like activity is the utilization of O₂ as the primary electron acceptor. Absorption of light of energy which is compatible with the bandgap of the material elevates the electrons to the conduction band and leaves holes in the valence band. The electrons are transferred to O₂ molecules, forming •O₂⁻ which oxidizes the organic substrate. This H₂O₂ creates a cascade of oxidative species by decomposing into •OH radicals. On the other hand, the holes either stabilize the intermediates or oxidize the substrate. The efficacy of this process is based on the ability of PNZs to rapidly transfer the electrons to O₂ and prevent the charge recombination (Jiang et al. 2025; Kapila et al. 2023; Wu et al. 2019).

Phosphorous modulated Carbon dots (P-CDNs): Incorporation of phosphorous onto carbon dots in the form of phosphoric acid generates defects for π-electron and functionalize phosphate. This will enhance their electron efficiency and promote oxidase like activity demonstrated through the oxidation of TMB. The performance of P-CDNs was 3.25 folds enhanced than CDNS (Jiang et al. 2025).

### Catalase (CAT) like activity

Photonanozymes perform catalase (CAT) like activity by disintegrating H₂O₂ to yield water (H₂O) and oxygen (O₂). This attribute offers self-oxygenation of the catalytic process and defense against oxidative stress. Generation of two electron/hole pairs initiates the reaction. Single substrate H₂O₂ when attached to PNZs, half of the molecules undergo oxidation and half of the molecules undergo reduction generating subsequent products. The electrons perform reduction and produce H₂O. Concurrently, the holes oxidize the remaining half reaction and form O₂. Overall, the net reaction is 2H₂O₂ → 2H₂O + O₂. Light irradiation elevates the reaction rate of this cycle by supplying charges, active site regeneration and lowering the activation energy mediated by nucleophilic attack of H₂O₂ on a metal site or the formation of a metal-peroxo intermediate bridge (Leng et al. 2025; Xu et al. 2022).

Zinc oxide Nanoparticles (ZnO NPs): They demonstrate catalase like activity with upconversion properties for photodynamic therapy (PDT). These ZnO NPs carry Chlorin e6 (Ce6) which is a photosensitizer and form ZnO-Ce6 NPs. They achieve deep penetration by converting their absorption spectrum from 808 to 401 nm to excite Ce6. They catalyze the decomposition of hydrogen peroxide to produce oxygen within tumors to eliminate hypoxia leading to antitumor immunotherapy. In the absence of light irradiation ZnO and ZnO-Ce_6_ NPs cause ferroptosis of tumor cells (Wang et al. 2024).

### Superoxide dismutase (SOD) like activity

The antioxidant function of PNZs is mediated by their potential to perform like superoxide dismutase (SOD) enzyme. Light irradiation enhances the reaction rate of dismutation of superoxide anion radicals (•O₂⁻) to molecular oxygen (O₂) and hydrogen peroxide (H₂O₂). This redox reaction is mediated by photogenerated electrons and holes in which one •O₂⁻ radical is reduced to H₂O₂ (by electrons) and the other radical is oxidized to O₂ (by holes). The overall reaction is presented as: 2•O₂⁻ + 2H⁺ → O₂ + H₂O₂. In this reaction the PNZs act as an electron shuttle which cycles the electrons between reduced and excited states. Along with shuttling, the light absorption by PNZs also boosts the reaction rate significantly. This light-induced charge separation ensures a rapid and continuous supply of redox mediators. This prevents the saturation of active sites and enhances the turnover frequency of PNZs as compared to their capacity to perform in the dark. This system is integral to augment the oxidative stress in biological and environmental samples (Gao et al. 2023; H. Zhao et al. 2021a).

Carbon dot nanozymes: The carbon based nanozymes display superoxide dismutase (SOD) like activity because of their ability to capture superoxide anions. Their activity is based on the hydroxyl and carbonyl groups of superoxide anions. They have vast applications in the treatment of oxidation related diseases (like ischemic stress) by the oxidation of damaged cells (Gao et al. 2023).

### Nitroreductase-like mechanism

The nitroreductase like activity of PNZs is mediated by the reduction of nitroaromatic compounds to their corresponding amino derivatives at the cost of two electrons without the need of flavoprotein cofactor NAD(P)H. The nitro reductase-like activity of photonanozymes is a light-driven catalytic process that mimics natural nitroreductase. enzymes by facilitating the reduction of nitroaromatic compounds to their corresponding amino derivatives. As the photons are harvested which exceed the bandgap, PNZs generate hot carriers and induce LSPR effect. The nitroaromatic substrates are pre-organized and form hydrogen bonds with the surface defects. The two hot electrons occupy the π orbital of the nitro group and form nitroso (Ar–NO) and hydroxylamino (Ar–NHOH) intermediates which transform into reduced amino product (Ar–NH₂) (R. Liu et al. 2021b; Y. Wu et al. 2024b).

Glutathione modified gold nanoclusters (G-AuNCs): The nanoclusters under visible light illumination catalyze the reduction of nitrobenzene to aniline. The photoinduced electron is transferred from excited Au NCs to nitro aromatic substrate producing radical intermediates which undergo reduction sequentially. The nitro group (-NO₂) is first reduced to a nitroso group (-NO), then to a hydroxylamine (-NH_2_OH), and finally to the amine (-NH₂), paralleling the stepwise reduction pathway of natural nitroreductases. The reaction is highly selective and biocompatible, enabling its application in prodrug activation within cellular environments. This photonanozyme system thus offers a promising strategy for light-controlled, site-specific prodrug activation in therapeutic contexts (R. Liu et al. 2021b).

### DNA-cleaving nuclease-like activity

In this activity PNZs mimic the activity of natural nuclease which cleaves the DNA molecules by hydrolysis or oxidative cleavage of phosphodiester bonds of the DNA backbone. The mechanism proceeds via an oxidative pathway rather than direct hydrolysis bypassing the production of ROS. The PNZs under light irradiation produce electron–hole pairs. The photogenerated holes oxidize surface adsorbed water molecules or hydroxide ions to hydroxyl radicals (•OH). These hydroxyl radicals (•OH) react with the deoxyribose sugar moiety in the DNA strand taking their hydrogen atoms and create a carbon-centered sugar radical intermediate. This radical intermediate reacts with molecular oxygen and fragments the sugar which cleaves the phosphodiester backbone resulting in single-stranded or double-stranded breaks. The efficiency of this system is dependent on the existence of ROS and DNA molecules in close proximity. This can be achieved by the electrostatic interaction between positively charged nanozyme surfaces and negatively charged DNA phosphate groups (Jiao et al. 2025; Li and Zhao 2025).

Copper complex and graphene quantum dots: Copper complexes contain aromatic ligands when conjugated with graphene quantum dots having similar size, they enhance the nuclease activity of copper complexes. The GQDs reduce the copper ions and accelerate their reaction with oxygen and produce superoxide anions which oxidize DNA molecules. These conjugates can have wide applications in anticancer therapeutics with high DNA selectivity (Zheng et al. 2014).

### Fatty-acid photodecarboxylase (FAP)-like mechanism

Photonanozymes perform fatty acid photodecarboxylase (FAP) like activity by utilizing light irradiation as a catalyst for the decarboxylation of free fatty acids into long chain hydrocarbons (n-alkanes) required for the synthesis of biofuels. The mechanism of action of PNZs follows the reaction pathway containing an intermediary radical of natural FAP. Light irradiation generates charge carriers (high energy electrons and holes). The holes (h +) oxidize the carboxy group (R-COO-) of the attached fatty acid and transform it into a carboxyl radical (R-COO•). In order to attain stability, the unstable carboxyl radical loses carbon dioxide (CO2) and forms an alkyl radical (R•). This alkyl radical takes the hydrogen from the hydrogen donor present in the vicinity (most often the solvent or substrate in the surroundings) to make a stable alkane product (R-H). The PNZs provide a platform which attaches the fatty acid substrate, pre-organizes it, produces the oxidizing species via photo illumination and stabilizes the reactive radical intermediates to inhibit the side reactions of radical coupling (Heyes et al. 2020; Spacey et al. 2025). Fe^3^⁺@TiO₂-based photonanozymes: In PNZs, TiO₂ exists in a pristine state which is the purest form without any impurities. It has a wide bandgap of ~3.2 eV which makes it absorb UV radiations and get activated. Doping of Fe^3^⁺ induces defects in theTiO₂ lattice (create oxygen vacancies) which reduce the bandgap energy and light absorption is extended to the visible light range. Exposure to visible light transfers the electrons from the mid gap (energy level of dopant or oxygen vacancies) to the conduction band. While the holes are localized to the surface of the TiO₂ lattice. Attachment of fatty acid substrate like palmitic acid (C16) onto the TiO₂ surface through a carboxylate group leads to its decarboxylation mediated by hole transfer. To stabilize the newly synthesized carboxyl radical, CO₂ is released. Dopant sites or oxygen vacancies capture the CO₂ molecule from the radicals and prevent the catalyst poisoning. This results in the efficient production of pentadecane (C15), a direct hydrocarbon fuel from the renewable fatty acid feedstock (Tan et al. 2023).

Table 2 depicts the diverse enzyme mimicking potential of photonanozymes which is more than functional replication of natural enzymes. They represent a basic rediscovering of catalysis where light is the sole driver of chemical transformation. They redefine the relationship between energy input and catalytic output regulated by the programmable gating of wavelength, intensity and duration of the reaction pathway. Collectively, these attributes induce a catalytic intelligence among PNZs which under different wavelengths orchestrate oxidation, reduction, degradation or synthesis. They fundamentally transform static catalysts into responsive, adaptive systems generating desired outcomes. The enzyme kinetics for nuclease like activity is not specified because graphene oxide (GO) acts as a heterogeneous catalyst instead of a classical enzyme. Reaction is occurring over the surface of GO rather than the formation of anenzyme-substrate complex. The DNA adsorbs the surface, photocatalytic reaction occurs, and the product desorbs. Therefore, Michaelis–Menten kinetic parameters do not adequately describe the kinetic parameters of nuclease-like DNA cleavage activity (Zhang et al. 2020a, b).

**Table 2:** Photonanozymes as multifunctional catalytic platform

Enzyme-like Activity	Enzymatic Reaction	Substrate Scope	Kinetic parameters		Activation wavelength (nm)	References
			Km (mM)	Vmax (µM min⁻^1^)		
Peroxidase-like (POD)	H₂O₂ + electron donor → oxidized donor + H₂O	H₂O₂; TMB; OPD	7.16	1.80	410	(Lin et al. 2022)
Oxidase-like (OXD)	O₂ + electron donor → oxidized donor + H₂O₂/•O₂⁻	O₂; TMB	0.65	2.25	420	(Zhao et al. 2025)
Catalase-like (CAT)	2H₂O₂ → 2H₂O + O₂	H₂O₂	0.63	19.6*	808	(Yang et al. 2024)
Superoxide Dismutase-like (SOD)	2•O₂⁻ + 2H⁺ → O₂ + H₂O₂	•O₂⁻	18.78	58.90	808	(Kang et al. 2020)
Nitroreductase-like	Ar–NO₂ + 2e⁻ + 2H⁺ → Ar–NH₂ + H₂O	Nitrobenzene	5.9 ± 1.4	150 ± 20*	450	(R. Liu et al. 2021b )
Nuclease-like (DNA-cleaving)	DNA phosphodiester bond → fragmented DNA	DNA molecules	-	-	365 & 450	(Zhang et al. 2020a, b)
Fatty Acid Photodecarboxylase-like (FAP)	R–COOH → R–H + CO₂	Free fatty acids	203.19	93.0 ^*^	410	(Tan et al. 2023)

^*^Unit is standardized to µM min⁻^1^

Photonanozymes conduct enzyme-like functions through an extensive pathway of photophysical and photochemical steps including light absorption, charge separation, substrate pre-organization, and bond activation by radical or high-valent intermediates, along with fast regeneration. The gating of these steps with respect to wavelengths, intensity, or external fields renders the system as programmable catalysts for environmental, biomedical, and energy applications.

Despite their versatile enzymatic applications, photonanozymes suffer practical limitations. While performing peroxidase and oxidase like activity, photonanozymes generate non-specific ROS causing off-target damages in biological systems specifically. The non-specific off target effect can be minimized via interventional photodynamic therapy. Moreover, cell membrane coated nanoparticles boost the confined generation of ROS at a specific tumor site (Duo et al. 2022).

The catalase like activity is hindered by the low levels of endogenous H₂O₂ within the tumor and creates a hypoxic environment hence, facilitating tumor growth. This challenge is overcome by incorporating MnO₂ nanozymes having intrinsic catalase like activity which produce O₂ and diminish the endogenous tumor hypoxia (Chen et al. 2025).

These multifaceted challenges are addressed through smart strategies integrating biomimetic membrane coating, interventional light delivery and standardized evaluation protocols to help unlock the maximum potential of PNZs in precision medicine and sustainable catalysis. Furthermore, the versatile behavior of PNZs in showing their activity as various enzymes has widened their application in diverse disciplines like biomedical diagnostics, environmental remediation, cancer therapy, nitrogen fixation and biofilm inhibition.

## Applications of PNZs

**Fig. 5:**
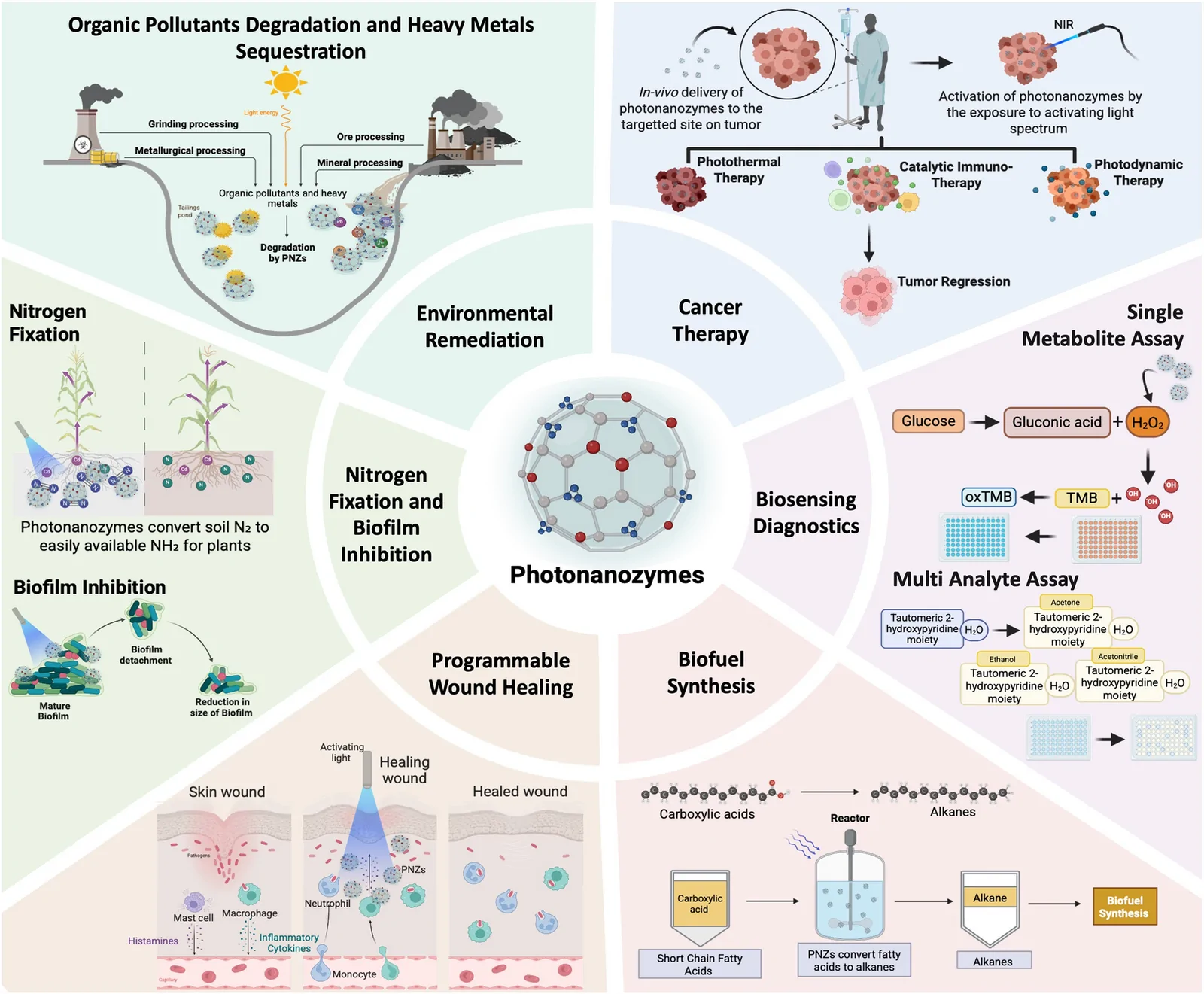
Applications of photonanozymes: Environmental remediation, biosensing diagnostics, cancer therapy, wound healing, nitrogen fixation and biofuel synthesis performed by the virtue of multiple-enzyme like activity of PNZs

Photonanozymes through their ability to perform enzyme-like catalysis mediated through photon harvest widen their application in versatile platforms like sensing, therapeutics, environmental remediation as presented in Fig. 5.

### Environmental remediation

Photocatalytic degradation of organic pollutants by photonanozymes is a multistage light-driven process using semiconductor/plasmonic excitation to generate ROS for mineralizing pollutants into CO₂ and H₂O. CeO₂/ZnO 3D nanoflowers featuring Z-type heterojunction have enhanced visible-light absorption, suppressing charge recombination and efficiently degrading tetracycline, rhodamine B, ciprofloxacin and methylene blue (Bekele and Alamnie 2025; Durodola et al. 2024; Huang et al. 2024; Swaminaathan et al. 2024).

Heavy metal sequestration by photonanozymes functions as optical transducers and redox sponges for heavy metal sequestration simultaneously. They transform toxic metal ions into detectable optical/electrical signals and reduce them to less toxic forms via photo-generated electron transfer. Their high surface area and functional groups mediate strong binding, enabling efficient sensing and removal of metal ions. The fluorescence spectrum of N doped carbon dots selectively enhanced in the presence of Hg2 + ions with strong pH responsiveness and temperature dependency. These characteristics define their applications for pH sensing, mercury ion detection and nano thermometry. (Fhoula et al. 2025; Unnikrishnan et al. 2021; Yan et al. 2020).

Photonanozyme-mediated photocatalytic degradation and heavy metal sequestration face several common challenges that impede their real-world application. Catalyst recovery remains a primary obstacle; while magnetic composites have been explored for easy recovery, the insufficient catalytic activity and non-recyclability of nanozymes are major hurdles for water purification. Even when recovery is achieved, activity often drops significantly after multiple cycles (Ahmad et al. 2025). Metal leaching poses another critical concern: metal-based nanozymes can release toxic ions under operational conditions, with one study reporting Fe leaching up to 420 ppb during catalytic reactions (Cao et al. 2023).

The integration of photocatalytic degradation and heavy metal sequestration within a single photonanozyme platform offers a compelling synergistic advantage. A unified system can simultaneously oxidize organic pollutants via photo-generated ROS while reducing toxic metal ions through photo-generated electrons, achieving comprehensive water decontamination in a single treatment step (Shi et al. 2025).

Furthermore, the sequestered metal ions can sometimes serve as co-catalysts, enhancing the photocatalytic degradation efficiency. However, this synergy is constrained by several limitations. The concurrent photochemical and chemical reactions may compete for charge carriers, thereby diminishing overall efficiency. Moreover, the necessity for periodic replenishment of hydrogen peroxide escalates both operational costs and safety risks (Wang et al. 2023).

To address these challenges, future design strategies should prioritize engineering closed-loop peroxide supply systems, leveraging machine learning to fine-tune nanozyme properties, and establishing standardized synthesis protocols. Immobilization of photonanozymes on robust membrane reactor supports has demonstrated potential in achieving superior stability with negligible metal leaching. These advancements, coupled with rigorous biosafety evaluations, are critical for transitioning photonanozyme technology from laboratory-scale demonstrations to practical, large-scale environmental remediation applications (Yu et al. 2025; Yuan et al. 2025).

### Biomedical diagnostics

Glucose and metabolite assays are performed by coupling the metabolite specific oxidase enzymes with their light enhanced peroxidase like activity. The colorimetric signals produced by the chromogenic products are amplified by PNZs' light enhanced efficiency with the concentration of metabolite. This combination of enzymatic specificity and nanozymatic amplification creates a highly sensitive and robust detection platform. CeO₂–MoS₂ nanozymes integrated with lactate oxidase enable electrochemical monitoring of sweat lactate, wirelessly transmitting voltage signals to a smartphone application (Ding et al. 2021; Patel et al. 2024; Qian et al. 2021; Weng et al. 2024).

Multi-analyte sensing assays incorporate a differential sensing tool in which an array of photonanozymes variants having specific physiochemical properties produce distinctive response patterns upon exposure to analytes. Algorithms designed to recognize the patterns decode them for complex sample analysis. As observed in excited state proton transfer (ESPT) sensors made of zinc-MOF nanosheets based on this approach showing fluorescence in water and quenched in the presence of organic solvents. This strategy is powerful for analyzing complex samples where specific receptors are indistinguishable (Chang et al. 2025; Yin et al. 2024).

Fluorescence immunoassays of PNZs are composed of specific antibody-antigen detection coupled with PNZs enzyme-like activity. The PNZ is conjugated to the recognizing antibody which forms an immunocomplex embedded over a solid platform. The complex containing PNZ is exposed to light irradiation of a specific spectrum which is compatible with the PNZs absorption band. Light illumination exponentially increases the PNZs catalytic turnover rate hence, revealing their POD like activity which converts non-fluorescence probe to highly fluorescent product. This offers a dual advantage by lowering the detection limit and enhancing the sensitivity compared with enzyme-based immunoassays (Liu et al. 2016; Tan et al. 2019; Zhou et al. 2022).

Conventional enzyme-based assays come with limitations of poor stability, denaturation and restricted signal amplification whereas, photonanozymes overcome these barriers through light triggered catalytic enhancement and multiplexed detection. They provide real time monitoring in complex biological matrices through the integration of enzyme-like activity with distinct recognition pattern coupled with antibody specific signal amplification which is challenging with the traditional enzyme-based immunoassays. Consequently, photonanozymes represent a paradigm shift in biosensing, transforming labor-intensive, laboratory-bound procedures into portable, user-friendly, and highly precise diagnostic tools for point-of-care applications.

The translation of photonanozyme-based biosensors from proof-of-concept to point-of-care (POC) devices is fundamentally constrained by a convergence of scientific, manufacturing, and regulatory hurdles. Unlike conventional laboratory settings, POC devices must withstand ambient light variations, operator variability, and complex biological matrices, where the "catalytic promiscuity" of inorganic surfaces leads to non-specific reactivity with interfering metabolites (Said 2026). A critical challenge lies in reproducibility and scalability, as high-performing photonanozymes are typically synthesized via microscale laboratory routes that fail to translate into industrial manufacturing, where maintaining uniform defect structures and catalytic stability is exceedingly difficult (Garg et al. 2026). Until these hurdles are systematically addressed through coordinated efforts in materials engineering, process optimization, and regulatory science, photonanozyme-based biosensors will remain confined to the laboratory rather than realizing their transformative potential in real-world diagnostic applications.

### Cancer therapy

Photodynamic therapy, a minimally invasive and selective cancer strategy, uses PNZs in tumors to generate singlet oxygen (Type II) and ROS (Type I) via photoactivation. These ROS cause irreversible oxidative damage, inducing cancer cell death. This localized approach spares healthy tissue. The Ag–TiO₂ nanorods demonstrate the ROS mediated cytotoxicity against breast cancer cells and 90% bactericidal activity specifically against *E. coli* and Staphylococcus aureus. (Cao et al. 2025; Hou et al. 2022; Sadati et al. 2025; Shoab Ali et al. 2024; Zheng et al. 2025).

Photothermal-catalytic (PTT-CDT) therapy synergistically combines localized hyperthermia with chemodynamic oxidative stress. NIR irradiation triggers PNZs to generate heat and convert endogenous H₂O₂ into toxic •OH radicals within the tumor microenvironment. MoS₂ nanosheets co-delivering curcumin and erlotinib exemplify this approach, enabling spatiotemporal control to minimize damage to healthy tissues. (Chen et al. 2023a, b; Naik et al. 2025; Sultana et al. 2025; R. Xu et al. 2025a).

Catalytic immunotherapy converts the immunosuppressive tumor microenvironment into an immunogenic hub by decomposing H₂O₂ into O₂ to relieve hypoxia and promote T-cell and NK-cell infiltration. This induces immunogenic cell death and durable, metastasis-targeting immune memory. MnO₂ nanoparticles exemplify this with effective photothermal ablation and MRI capability (Kong and Chen 2022; J. Liu et al. 2021a; X. Wu et al. 2024a; Xu et al. 2025a, b).

The true potential of photonanozyme-based cancer therapy lies in the synergistic integration of PDT, PTT-CDT, and catalytic immunotherapy within a single nanoplatform. This multimodal approach addresses the inherent limitations of individual modalities. The combination of these therapeutic modalities leads to a cascade of synergistic effects: heat from PTT accelerates Fenton-like reactions in CDT, ROS from PDT activates immune pathways, and immunotherapy ensures durable antitumor memory. Moreover, the spatial and temporal control offered by NIR light further amplifies these synergies, enabling precise, localized treatment while minimizing systemic toxicity (Ding et al. 2025; Feng et al. 2025; Sadati et al. 2025).

Despite the immense potential of photonanozyme-based cancer therapies, several critical limitations hinder their clinical translation and widespread application. These challenges span biological, technical, and manufacturing domains. Elimination from the Body: The long-term fate and clearance of photonanozymes remain significant concerns. Inorganic nanomaterials tend to accumulate in the reticuloendothelial system (liver and spleen), raising concerns about chronic toxicity and organ damage. Their biodegradation pathways are often slow, leading to persistent retention and potential long-term side effects (Mohammadpour et al. 2019; Pizzoli et al. 2025). Side Effects: Non-specific generation of ROS in healthy tissues remains a major safety concern. Despite NIR-triggered activation, some photonanozymes exhibit background activity, leading to unintended oxidative damage. Furthermore, the photothermal effect can cause thermal damage to surrounding tissues if not precisely localized (Mishra et al. 2021; Zheng et al. 2025). Non-Specific Delivery: Efficient delivery of photonanozymes to the tumor site is a significant challenge. Issues such as poor targeting efficiency, insufficient accumulation via the enhanced permeability and retention (EPR) effect, and clearance by the mononuclear phagocyte system limit therapeutic efficacy and contribute to off-target effects (Xu and Fan 2026; Zelepukin et al. 2024). Allergic Reactions: The administration of photonanozymes, particularly those containing transition metals or organic ligands, can stimulate immune responses such as hypersensitivity reactions or immunogenicity, especially upon repeated administration (Huang et al. 2025; Shukla et al. 2016). Functionality: The functionality of photonanozymes is often compromised in the complex tumor microenvironment due to factors such as low endogenous H₂O₂ levels (limiting CDT), hypoxia (limiting PDT), and protein corona formation, which masks active sites (Zang et al. 2025). Large-Scale Industrial Production: The scalable manufacturing of photonanozymes with batch-to-batch reproducibility, controlled morphology, surface chemistry, and defect engineering remains a formidable challenge. Complex synthesis protocols, high costs, and stringent quality control requirements hinder their transition from laboratory to clinical practice (Quintero‐Garrido et al. 2026).

The synergistic integration of PDT, PTT-CDT, and catalytic immunotherapy within photonanozyme platforms offers transformative potential for precision oncology; however, the clinical translation of these multimodal systems is critically hindered by challenges in long-term biocompatibility, tumor-specific delivery, and scalable manufacturing. Future research must prioritize the rational design of biodegradable, tumor-microenvironment-responsive photonanozymes with standardized, scalable synthesis protocols to bridge the gap between proof-of-concept and clinical application.

### Nitrogen fixation and biofilm inhibition

Nitrogen fixation and solar fuel synthesis by photonanozymes are a light-driven process which follows natural photosynthesis to convert by cleaving the strong triple bond in N₂ (N≡N). (Xia et al. 2025). The Oxygen-Vacancy-Rich BiOBr Nanoflakes contain surface oxygen vacancies that act as active sites. Under visible light, the vacancies efficiently trap electrons, which are transferred to the π* orbitals of adsorbed N₂ molecules, facilitating reduction to NH₃. Simultaneously, the material can catalyze H₂ evolution from water (Li et al. 2015).

The antibacterial and antifouling effect of PNZs is induced through light triggering, self-regeneration combined with biofilm inhibition via contact killing. The PNZs produce ROS through their peroxidase like activity which oxidizes lipids present in the membrane of the adhering bacteria with denaturing proteins, damaging DNA and lysing the cells. An accompanied continuous oxidative barrage degrades adhesive extracellular polymeric substances (EPS) that form the biofilm matrix. The photocatalytic process often bestows superhydrophilicity to the surface, creating a physical hydration barrier that repels initial microbial attachment. This dual mechanism ensures the surface remains clean and bioactive (Hu et al. 2025; Yan et al. 2025; Zhao et al. 2024).

### Programmable ROS regulation (wound healing)

The mechanism of wound healing photonanozymes functions by the localized generation of ROS performing a dual role by inducing a bactericidal effect without damaging surrounding tissues and also promoting angiogenesis, fibroblast proliferation and collagen deposition which speed up the healing process (Cui et al. 2025; H. Zhang et al. 2025a). This has been observed in ROS modulating Ti₃C₂Tₓ-Se NPs (TS) Nanosheets presenting bactericidal catalase and superoxide dismutase activity designed for wound healing against Staphylococcus aureus and *E. coli*. They eliminate the bacterial infection and also remove the ROS hence, reducing the inflammation in the treatment of wound healing (Cui et al. 2025).

### Photodecarboxylation (biofuel synthesis)

The PNZs have been used to synthesize biofuels while imitating the activity of natural fatty acid photodecarboxylase enzymes. The photogenerated holes oxidize the carboxylate group (R-COO⁻) of the attached carboxylic acid (free fatty acid) into hydrocarbon biofuels. The PNZs surface properties are crucial for adsorbing and pre-organizing the fatty acid substrate, facilitating efficient electron transfer, and stabilizing the radical intermediates to prevent undesirable side reactions (Sorigué et al. 2021; Tan et al. 2023). Defect-rich TiO₂ Nanozymes: Oxygen vacancies in TiO₂ create mid-gap states that enable visible light absorption. The vacancies act as adsorption sites for fatty acids. Under visible light, holes localized near these vacancies initiate the decarboxylation of palmitic acid (C16:0) to pentadecane (C15), a diesel-range hydrocarbon (Tan et al. 2023).

## Conclusions

Photonanozymes drive a shift in biomimetic catalysis by their distinct ability to harness light as an external stimulus for spatiotemporally regulated enzyme-like activity. By the integration of a light harvesting domain engineered with an active site within the nanoenvironment, PNZs exhibit remarkable versatility across peroxidase, oxidase, superoxide dismutase, catalase, nitroreductase and fatty acid photodecarboxylase pathways. The PNZs perform as a reliable strategy in biomedicine, environmental remediation and sustainable energy conversion by the virtue of synergistic interplay among molecular architecture, composition and microenvironmental parameters. Indeed, with these developments, several shortcomings hinder their clinical applications and industrial scalability. The in vivo toxicological analyses for PNZs to understand immunogenicity, biodistribution and long-term clearance need to be explored. Development of biodegradable constructs having regulated degradation kinetics to avoid persistent accumulation in the body specifically in the kidneys and liver.

Limited penetration of activating light in the biological tissues serves as an additional challenge in the application of PNZs. Up-converting the nanoparticle-coupled PNZs which transform deep penetrating near infrared (NIR) light (808 nm) to UV (visible emission) that enable their activation in deep tissues. This can be coupled with interventional optical fiber therapy which enhances their site-specific delivery.

The mechanistic interplay of PNZ activation which include atomic interaction at the defect sites, carrier dynamics and pre-organization of substrate need further exploration. This can be achieved by opting for operando techniques in the characterization (i.e. time resolved X-ray absorption, transient absorption spectroscopy). Coupling these techniques with machine learning models trained over defect engineering catalyst libraries. These AI driven approaches can help predict the structure activity relationships for targeted ROS generation. Accelerating rational design over empirical trial and error method.

Furthermore, their industrial scale application requires their conversion into recyclable smart materials like immobilized photoreactor coating or magnetic core composites working at a macroscopic scale. They will enable their continuous flow catalysis with catalytic loss and energy input. Standardizing these synthesis protocols is necessary for batch-to-batch reproducibility. A phased roadmap, beginning with systematic operando studies to establish mechanistic foundations, followed by AI-driven discovery cycles to accelerate the identification of biodegradable, NIR-responsive PNZ candidates, and culminating in pilot-scale validation in membrane reactors, will provide a cohesive translational pathway.

Addressing these integrated challenges helps in establishing a strong and cohesive framework of PNZs from proof of concept to practical tools. The strategy based on real-time characterization, rational material engineering, and AI guided predictive synthesis will position PNZs as a precise catalyst hence, unlocking their applicability in medicine, environmental sciences and sustainable catalysis.

## Data Availability

This is a review article that synthesizes and critically evaluates previously published scientific literature. No new primary data, experimental results, or original datasets were generated or analyzed in support of this manuscript. All data referenced in this review are available in the respective published articles cited within the reference list and their supplementary materials.
